# Self-Interaction Chromatography of mAbs: Accurate Measurement of Dead Volumes

**DOI:** 10.1007/s11095-015-1758-3

**Published:** 2015-08-13

**Authors:** S. H. M. Hedberg, J. Y. Y. Heng, D. R. Williams, J. M. Liddell

**Affiliations:** Surfaces and Particle Engineering Laboratory, Department of Chemical Engineering, Imperial College London, London, UK; R&D Group, FUJIFILM Diosynth Biotechnologies, Billingham, UK

**Keywords:** dead volume, mAbs, protein-protein interactions, second virial coefficient, self-interaction chromatography

## Abstract

**Purpose:**

Measurement of the second virial coefficient B_22_ for proteins using self-interaction chromatography (SIC) is becoming an increasingly important technique for studying their solution behaviour. In common with all physicochemical chromatographic methods, measuring the dead volume of the SIC packed column is crucial for accurate retention data; this paper examines best practise for dead volume determination.

**Method:**

SIC type experiments using catalase, BSA, lysozyme and a mAb as model systems are reported, as well as a number of dead column measurements.

**Results:**

It was observed that lysozyme and mAb interacted specifically with Toyopearl AF-Formyl dead columns depending upon pH and [NaCl], invalidating their dead volume usage. Toyopearl AF-Amino packed dead columns showed no such problems and acted as suitable dead columns without any solution condition dependency. Dead volume determinations using dextran MW standards with protein immobilised SIC columns provided dead volume estimates close to those obtained using Toyopearl AF-Amino dead columns.

**Conclusion:**

It is concluded that specific interactions between proteins, including mAbs, and select SIC support phases can compromise the use of some standard approaches for estimating the dead volume of SIC columns. Two other methods were shown to provide good estimates for the dead volume.

## Introduction

Monoclonal antibodies (mAbs) have successfully been used in clinical trials for treatments of several cancer and autoimmune diseases (1–3) and have lately become the fastest growing segment of the biopharmaceutical industry (4, 5). Currently 48 therapeutic monoclonal antibodies are approved or in review by the EU or the USA. They are represented as the second largest biopharmaceutical category under investigation as therapeutic drugs and have therefore become vitally important for modern medicine (6). The manufacture of mAbs in a cost-effective and reliable fashion still remains a major international challenge in spite of the success antibody therapeutics have had in the pharmaceutical industry (7, 8).

The current understanding about the way mAbs behave in different solution conditions, and the specifically the effects of solution conditions on formulation stability is still not fully understood, especially in terms of the mechanisms leading to aggregation and their long-term aggregation stability (9, 10). Therapeutic proteins such as mAbs have complex structures and often have relatively large molecular sizes, so when they are exposed to certain solution conditions they are prone to physical instability and tend to aggregate (11, 12). In order to minimise the problems associated with aggregation in bioprocessing, the ability to predict, for complex proteins such as mAbs both their propensity to aggregation as well as aggregation rates would be a valuable tool in process and formulation development (13). Another key challenge in protein formulation is viscosity, especially when proteins are present in higher concentrations as is the case of therapeutic drugs requiring high dosages (14).

Protein-protein intermolecular interactions are known to be involved in protein solution aggregation behaviour (15). However, the exact mechanisms leading to protein aggregation are still not fully understood (15–17). The osmotic second virial coefficient (B_22_) is a fundamental physiochemical property that describes protein-protein interactions in solution and can be a useful tool for predicting the aggregation propensity of proteins as well as other solution conditions such as those suitable for protein crystallisation. Quigley and Williams (18) have reported an extensive B_22_ study over a range of proteins in aqueous solutions across a wide range of pH’s and salt concentrations. This work showed that the osmotic second virial coefficient, B_22_, was an accurate descriptor of protein aggregation behaviour in terms of hydrodynamic radius for a number of model proteins.

B_22_ comes from the virial equation of state, which describes the behavior of non-ideal gases (real gases). A solution thermodynamic model of the intermolecular forces between gas molecules can also be applied analogously to solvent molecules interacting with proteins in solution, and is known as the McMillan and Mayer theory (19). B_22_ describes non-ideal solution behaviour and can be used to quantify intermolecular forces between two protein molecules (20). B_22_ describes the direction and magnitude of overall intermolecular forces, in which positive B_22_ values represent net repulsive forces where protein-solvent interactions are favoured, while negative B_22_ values indicate net attractive forces where protein-protein interactions are favoured, which cause aggregation to occur.

The first experimental techniques employed to determine B_22_ were membrane osmometry and static light scattering, though both of these techniques require several solution concentrations to determine a single B_22_ value. Additionally, these techniques can be time consuming and require relatively large amounts of protein for reliable B_22_ estimates (21). Self-Interaction chromatography (SIC) is a relatively new technique and was first reported by Patro and Przybycien (22) who used it to predict protein aggregation. SIC involves immobilising a target protein onto a solid state material which is usually a chromatographic stationary phase. The same protein will then be used within the mobile phase as well, and a pulse of protein (together with buffer) will be injected and eluted through the packed column which contains the immobilised protein. The elution behaviour, and specifically the elution volume for the protein which passes through the column packed with the immobilised protein are accurately measured, normally using a UV–Vis LC detector. The chromatographic measurement of the retention volume between the protein in the mobile phase and the protein immobilised on the stationary phases forms the basis of SIC.

This type of chromatography is based on weak affinity chromatography principles, but in this technique the same protein will both act as ligand and ligate. Tessier *et al.* (23) developed a model from the SIC method described by Patro and Przybycien (22), and an expression for the interaction between two protein molecules in solution in terms of the osmotic B_22_ as originally presented by Zimm (24) and McQuarrie (25) for determination of B_22_. The model is based on a statistical mechanics based analysis. The net retention volume data represents the protein-protein interactions, and is used together with the excluded volume contributions, the phase ratio and the number of immobilised protein molecules per unit area. Over the following decade, the technique continued to be developed and has been applied to a number of model and therapeutic proteins, many employing the same experimental immobilisation methods as described originally by Tessier *et al.* (23). SIC has more recently become a technique being used for studying therapeutic proteins. Payne *et al.* (26) were the first to use SIC for the determination of B_22_ for a therapeutic peptide that would not scatter light sufficiently to be studied using static light scattering (SLS). Le Brun *et al.* (27), Lewus *et al.* (28) and Ahamed *et al.* (29) have investigated B_22_ for monoclonal antibodies, reporting the effects of ionic strength and pH, but also the use of co-solvents such as PEG (28, 29) and temperature variations (27). Correlations of B_22_ with both solubility and crystallisation behaviour have been reported (30–36) as well as models created for best formulation conditions (37). Correlations between aggregation and B_22_ have also been reported using experimental techniques other than SIC (38). Another technique reported includes dynamic light scattering that can determine the protein-protein interaction parameter in quite a high-throughput manner and has also proved useful in formulation development (39).

### Determination of the Second Virial Coefficient

The dead volume is defined as the interstitial space between the particles in the column as well as any other volume in the column and tubing that is not packed. In order to determine B_22_, the dead volume or the volume required for a non-interacting molecule of the same size as the protein to pass through the SIC column needs to be accurately measured. The non-interacting molecule used for this measurement must be carefully chosen. Not only must this molecule not interact specifically with the immobilised protein or the phase it is immobilised on, but it should also sense the same volume within the stationary phase that would be sensed by a protein eluted via the mobile phase. So, for example, it cannot just be any non-interacting molecule such as acetone because protein molecules are not able to access as much of the pore space in the immobilised phase as can smaller molecules such as acetone. This size exclusion effect has been argued to be a reason not to use acetone retention volumes as a dead volume measure (23). In the literature, the dead volume has been determined using two main methods.

The first procedure evaluated by Tessier *et al.* (23) involved using a dead column, which is a SIC column prepared in the same way without immobilising the protein on the support, and measuring the retention volume of both acetone (V’_a_) and protein (V’_p_) passing through the dead column. The dead volume, V_0_, can then be estimated by:

1 $$ {V}_0 = {V}_a\left(\frac{V^{\prime }p}{V^{\prime }a}\right)-{V}_i $$

where *V*_*i*_ is the volume occupied by the immobilised protein molecules and V_a_ is the acetone retention volume in the immobilised column. For the latter calculation protein molecules are assumed to be spherical and the diameter is calculated from the molecular volume, described by Neal and Lenhoff (40).

The second experimental procedure for determining the dead volume was proposed by Binabaji *et al*. (41), which involved using dextran molecules of different molecular weights as the dead volume marker. They determined that the dextran standard that had the retention volume closest to the mAb of 142 kDa using a Superdex 200 column served as the non-interacting solute and would be then used to estimate V_0._ In their study this solute was a 50 kDa dextran MW standard and this dextran was injected onto the mAb immobilised column to directly determine V_0_.

Then B_22_ can be obtained by the equation:

2 $$ {B}_{22}=\frac{N_A}{M_W^2}\ \left({B}_{HS} - \frac{k^{\prime }}{\rho_s\Phi}\right) $$

where

3 $$ {k}^{\prime } = \frac{V_p - {V}_0}{V_0} $$

*k*′ is a chromatographic retention factor where *V*_*P*_ is defined as the retention volume of the protein in the protein immobilised column. *B*_*HS*_ in Eq. 2 is defined as the excluded volume or the hard sphere contribution defined by $\frac{16}{3}\pi {r}^3$ using the protein radius. *ρ*_*s*_ is defined as the number of immobilised molecules per unit area and obtained by dividing the concentration of immobilised protein by the porosity (0.811 for the 650 M resin) and the phase ratio (Φ) of one protein molecule. The phase ratio is given by A_s_ / V_0_ where A_s_ is the total accessible surface area, which is available to the mobile phase protein. This data was calculated by using inverse size exclusion chromatography data for different size dextran molecules for a variety of particles presented by DePhillips and Lenhoff (42).

B_22_ as specified above is dependent on the molecular weight of the protein, so in order to more accurately present the results and compare B_22_ results between different proteins the dimensionless second virial coefficient (B_2_) is used. B_2_ can be obtained from B_22_ through the following equation (43):

4 $$ {B}_2 = \frac{B_{22}{M}_W^2}{N_A{B}_{HS}} $$

The retention times for the protein and acetone injections on all column were determined using first moment (centre of mass) approach recommended by Quigley *et al.* (44). The retention times using this technique were compared to those determined by peak maximum. In cases where the chromatograms were Gaussian shaped the first moment analysis and peak maximum retention times/volumes will be coincident. For acetone injections the chromatograms were almost completely Gaussian, which resulted in similar results for both methodologies. However, for protein injections many chromatograms showed tailed peaks, confirming the importance of a first moment analysis as used here.

A key challenge that remains in evaluating B_2_ accurately is estimating the dead volume (41). This important topic has received limited attention, and in the previous literature very few details on how the dead volume in the dead columns were determined in terms of key variables such as solution conditions are reported (23). The objective of this work is to critically evaluate the techniques previously described for estimating the dead volume in SIC and, applying the best practise dead volumes corrections, so as to calculate B_2_ for a mAb in conditions where it is known to have poor solution stability.

## Materials and Methods

### Materials and Equipment

Experiments with the model proteins were performed with hen egg white lysozyme (crystalline white powder, EC 3.2.1.17), bovine liver catalase (lyophilised powder, EC 1.11.1.6), bovine serum albumin (BSA), (lyophilised powder, A-7638) all obtained from Sigma-Aldrich (Dorset, UK) and a monoclonal antibody of IgG1 type supplied by FUJIFILM Diosynth Biotechnologies which was highly purified (pI of 8.6 and molecular weight 144.5 kDa). The dextran molecular weight standards were obtained from Sigma-Aldrich. The 50 kDa standards (00891 and 31420) had a polydispersity index of <1.5. Potassium phosphate, NaBH_3_CN, dibasic and monobasic sodium phosphate, ethanolamine, HCl and NaOH were all purchased from Sigma-Aldrich (ACS or BioXtra grade). NaCl, Sodium acetate trihydrate, glacial acetic acid and acetone were obtained from Fisher Scientific (AR grade). Toyopearl AF-Formyl-650 M (08004) and Toyopearl AF-Amino-650 M particles (08002) were purchased from Tosoh Bioscience. For buffer preparation ultrapure deionised water (resistivity ~18.2 MΩ∙cm) was used. The pH of the buffers were adjusted with HCl or NaOH and monitored using a Mettler Toledo FiveEasy pH meter. All solutions were filtered using 0.22 μm bottle-top filters from Millipore in order to remove particulates before analysis.

Self-Interaction Chromatography (SIC) experiments were performed using an Agilent 1100 series liquid chromatograph (Agilent Technologies, Cheshire, UK). The model consisted of a binary solvent pump, an autosampler, with a sample injection valve, a thermo stated column compartment and two variable wavelength UV/Vis detectors, one placed before the column and one after. Having a UV detector both before and after the column enables determination of any protein being irreversibly bound to the column or protein being lost from the column due to denaturation. Buffers entering the system were filtered through inlet filters, and the dissolved air in the solvents was removed with a Phenomenex Degassex vacuum four-channel on-line degasser (Phenomenex, Torrence, CA). The liquid chromatography system was controlled and data was collected using Chemstation software version A.10.02 for HPLC systems (Agilent Technologies, Cheshire, UK). The particles with immobilised proteins, serving as the stationary phase, were slurry packed into an empty glass column, 100 mm long with an internal diameter of 6.6 mm from Omnifit with one fixed and one adjustable end piece pre-assembled with 25 μm PE frits (Fisher Scientific).

### Preparation of Dead Columns

The traditional dead column preparation consisted of packed columns prepared in the same way as a normal SIC without protein immobilised on them. Other workers have not directly considered the possible specific interactions between the dead column materials and the target proteins. These specific interactions could arise from the covalent bonding that can occur between the resin and the proteins.

In past immobilisation procedures reported for lysozyme and mAb, the protein amine groups form covalent bonds with the formyl or aldehyde groups on the Toyopearl AF-Formyl particles. In the same way the amine groups available on the Toyopearl AF-Amino particles form covalent bonds with carboxyl groups or aldehyde groups on the proteins, which is the case for catalase and BSA.

The dead columns prepared in this study consisted of Toyopearl AF-Formyl particles and AF-Amino particles without immobilised proteins. At least two of each column type was prepared to test data reproducibility. The Toyopearl AF-Formyl columns were prepared both with and without the addition of ethanolamine and sodium cyanoborohydride, NaBH_3_CN, to see if the addition of these reagents produced any difference in results. The Toyopearl AF-Amino columns were prepared the same way without any addition of reagents.

### Immobilisation and Column Packing

The immobilisation of mAb were carried similarly to the process described by Tessier *et al.* (23). Three ml of Toyopearl AF-Formyl particles were first washed with 250 ml of deionised water on a glass frit with a 0.2 μm polyethersulfone membrane filter (Sigma-Aldrich) and then mixed with mAb at a concentration of 10 mg/ml in 0.1 M potassium phosphate buffer. Afterwards, 90 mg of NaBH_3_CN was added to the suspension and the reaction was left to proceed overnight on a rotary mixer. The next day the particles were washed with 0.1 M potassium phosphate buffer and the remaining active sites were capped using 20 mg of cyanoborohydride and 15 ml of 1 M ethanolamine. The reaction was finally allowed to proceed for at least 4 h on a rotary mixer.

The protein immobilised particles were then slurry packed into an empty column and washed *in situ* with 100 ml of 0.1 M potassium phosphate, then with approximately 100 ml of 1 M NaCl and once again with approximately 100 ml 0.1 M potassium phosphate. The washes were performed to remove any protein not covalently bound to the stationary phase and all the washes were collected (from the initial protein solution to the final washes *in situ*) in order to calculate the net amount of protein immobilised on the stationary phase using mass balance. The concentration of residual protein found in the washes was determined by UV spectroscopy at 280 nm and a BCA protein assay.

Before any SIC experiments with protein were performed, a 50 μl pulse of 2% *v*/*v* aqueous solution of acetone was injected through the column and the peak was analysed for symmetry. The aim is to obtain sharp Gaussian peaks to indicate that the column is properly packed without any channelling occurring. For the SIC experiments the same protein as immobilised on the column was dissolved in selected buffer solutions in glass vials in the autosampler and is referred to as the protein mobile phase. The buffer used for the protein mobile phase was always the same buffer as being pumped through the system.

The column was conditioned with buffer of the same pH as was going to be used in the experiments. Experiments were not initiated until the UV baselines from the detectors were stable. Before any injections were made an equilibration time of 30 min was allowed. An experimental run consisted of injecting a pulse of the protein mobile phase and monitoring the absorbance until all the protein had eluted through the column. All injections were repeated in triplicates to ensure reliability of the results. The injection sizes were 10 μl for the model proteins and 20 μl for the mAb, the injection concentrations were 10 mg/ml for lysozyme, BSA and mAb, and 3 mg/ml for catalase. The flow rate was 0.5 ml/min and the buffer conditions were 20 mM sodium acetate for pH 4.5–5.0 and 20 mM sodium phosphate for pH 6.0–8.0.

## Results and Discussion

### Dead Volume Measurement using a Dead Column

The initial experiments to measure the column dead volume (V_0_) were based on the use of a dead column that was packed with Toyopearl AF-Formyl particles without any immobilised protein, the currently recommended and the most practiced technique. The retention volume for a number of different proteins and acetone were measured and the V’_p_/V’_a_ ratio’s calculated. These experiments were run at a range of pH’s and NaCl concentrations. According to Tessier *et al.* (23) using a NaCl concentration >0.8 M should eliminate protein-surface interactions and thus protein injections at <0.8 M NaCl would show interactions in their chromatograms. Overall it presumed that the dead columns will provide the same information as ideal non-interacting molecules of the same size as the protein. In testing the dead column, a number of pH conditions were evaluated between pH 4.5 and 8.0.

Catalase and BSA showed no or minimal differences in the retention volume for the different pH conditions at 0.8 M NaCl. Lysozyme showed a slight increase in retention volume at pH 4.5 but the mAb showed quite an extensive increase in retention volume when this pH was reached. In general the conditions pH 6.0 and 8.0 showed the same retention volume for both lysozyme and mAb.

**Fig. 1:**
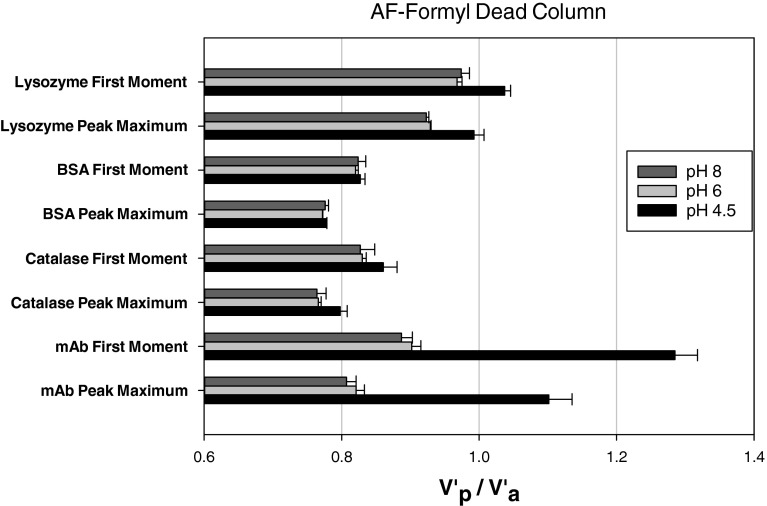
Protein Retention behaviour on a Toyopearl AF-Formyl dead column under different pH conditions (0.8 M NaCl).

Figure 1 shows that catalase and BSA have a retention behaviour that is independent of pH. The pH independent behaviour of these proteins is consistent with the hypothesis previously advanced that 0.8 M NaCl is able to shield the protein-surface electrostatic interactions (23). However, for mAbs, and a lesser extent lysozyme, there is a clear dependency of retention volume with pH. This observation is very significant. Firstly, it implies that electrostatic interactions between the proteins were not fully shielded by the 0.8 M NaCl of electrolyte present in the protein solutions. Whilst secondly, it shows that dead volume measurements can be both pH and protein-column dependent.

In order to confirm if this was a particle specific effect, another dead column was packed with Toyopearl AF-Amino particles and the same experimental method used. This column was used to minimise the direct interactions between surface groups that could lead to a covalent linkage.

**Fig. 2:**
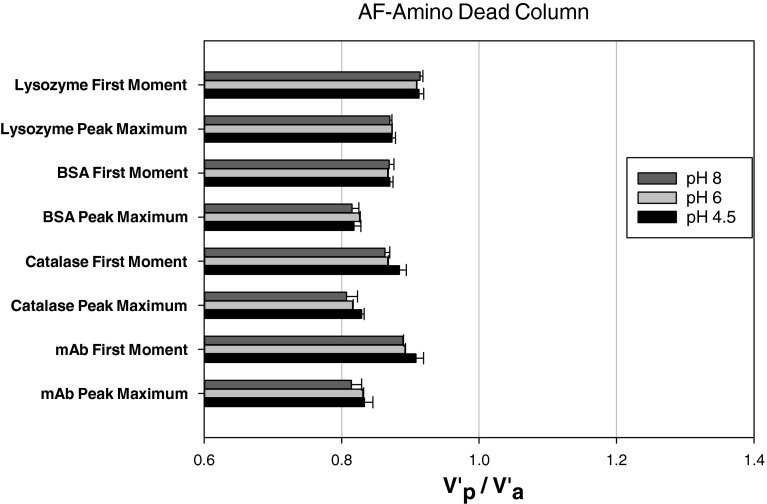
Protein retention behaviour on a Toyopearl AF-Amino dead column under different pH conditions (0.8 M NaCl).

Figure 2 shows that for all 4 proteins retention on the Amino dead column was constant and independent of pH, in contrast to that shown in Fig. 1.

The results from Figs. 1 and 2 show unequivocally that there can be specific interactions between the proteins and the dead column packing phase, and therefore the choice of dead column packing phase needs to proceed with care if it is to accurately represent the dead volume. Furthermore, in the case of larger injection sizes with high concentrations, there would also a concomitant risk of having protein irreversibly immobilised onto the column during the actual SIC experiments as proteins have previously been immobilised on packed columns in the presence of suitable reagents (45). Theoretically, during the immobilisation procedure for lysozyme and mAb the amine groups on the proteins form covalent bonds with the aldehyde groups on the Toyopearl AF-Formyl particles, and indeed such reactions may account for the data shown in Fig. 1 for lysozyme and mAb. However, though catalase and BSA can bind to the amine groups available on the Toyopearl AF-Amino particles, no evidence of such interactions was observed for these dead column experiments. It has previously been shown that protein immobilisation can be performed in pre-packed columns by slowly passing the protein through the column (45, 46). However, these injections are very small in size and in such cases the amount of protein that would be immobilised would be insignificant.

**Fig. 3:**
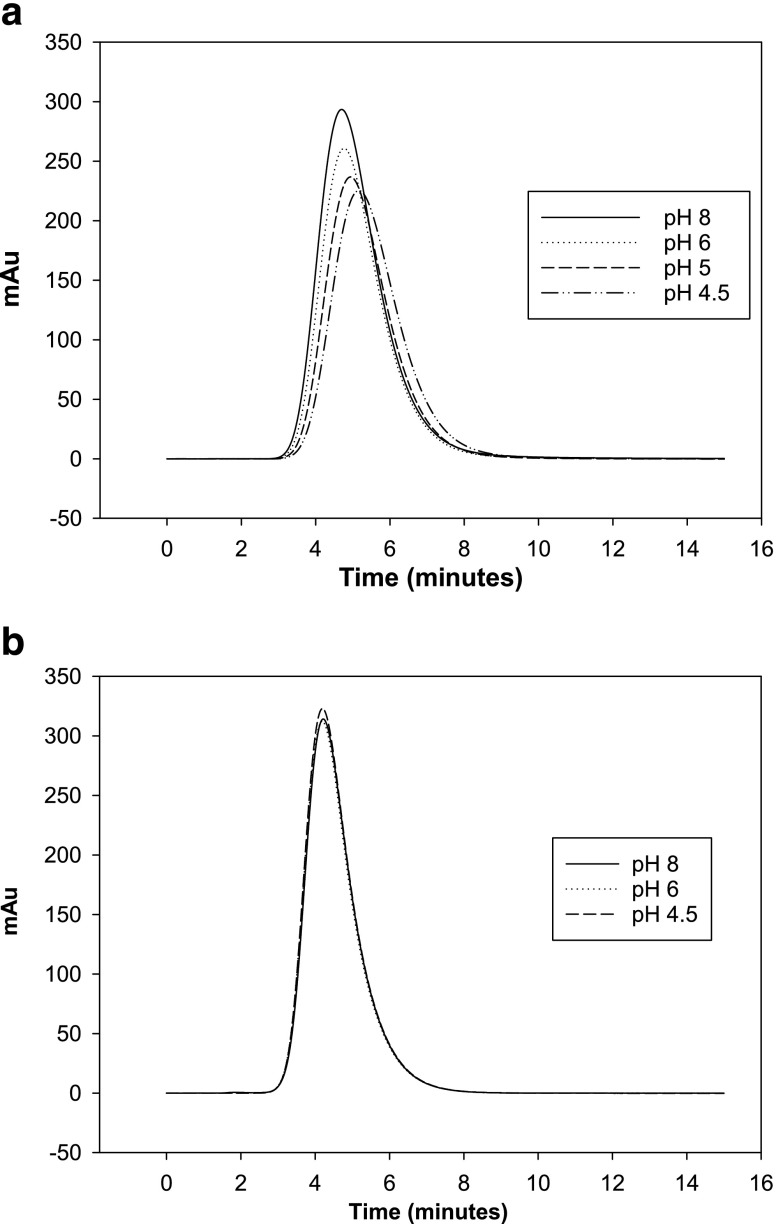
Chromatograms for lysozyme injections on a Toyopearl AF-Formyl column (**a**) and a Toyopearl AF-Amino column (**b**) under different pH conditions (0.8 M NaCl).

Figure 3a and b shows the effect of lysozyme injected on the two dead column. The peaks displayed in Fig. 3a shows a minimum shift in retention time for pH values ≤6.0 with no direct shifts seen in Fig. 3b. In Fig. 3a the shift in retention time (peak maximum) is from 4.70 to 5.16 min with decreasing pH, which causes V’_p_/V’_a_ on average to change from 0.97 to 1.04 (first moment) and from 0.92 to 1.00 (peak maximum). In Fig. 3b, V’_p_/V’_a_ is 0.91–0.915 (first moment) and 0.87–0.873 (peak maximum), which typify the errors found for different columns and injections in this study.

**Fig. 4:**
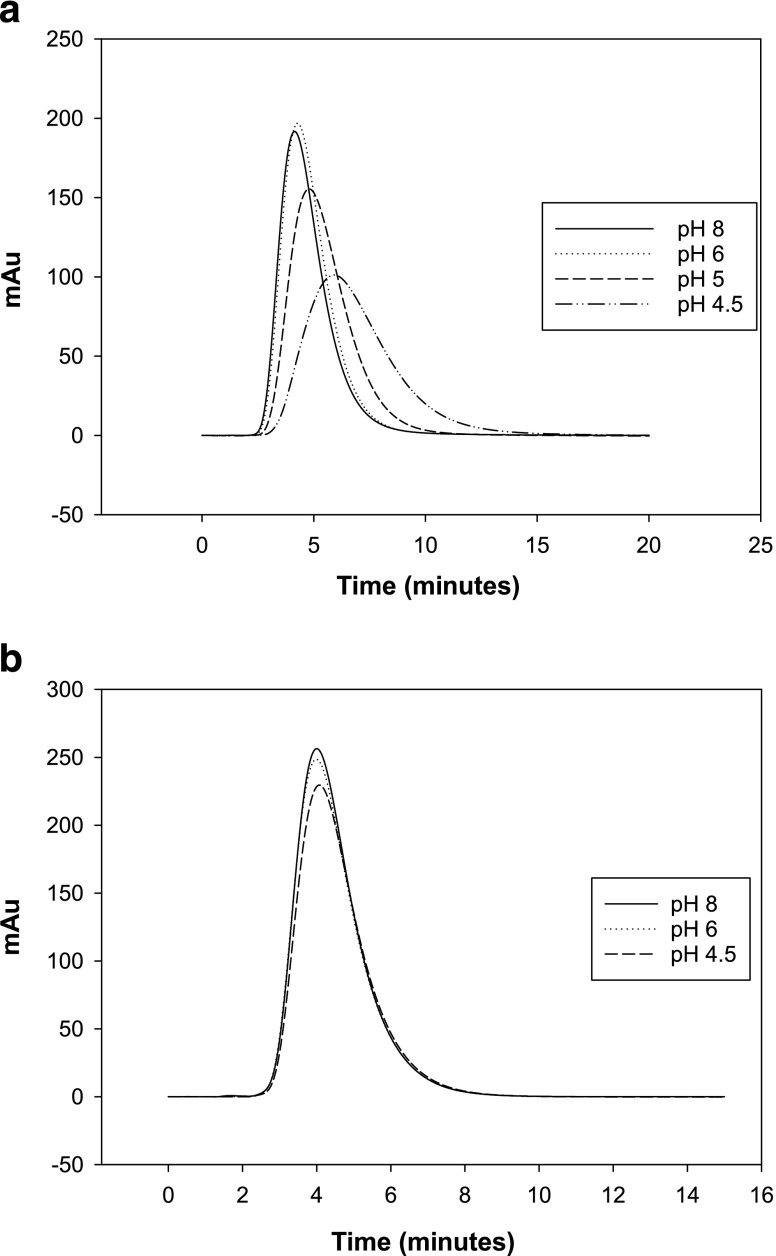
Chromatograms for mAb injections on a Toyopearl AF-Formyl column (**a**) and a Toyopearl AF-Amino column (**b**) under different pH conditions (0.8 M NaCl).

Figure 4a shows a more significant shift in the SIC chromatograms for mAbs compared to those shown in Fig. 3a. The mAb chromatograms are highly dependent on the solution pH when eluted through a AF-Formyl column. Figure 4b shows that the mAb retention is independent on pH, in a similar fashion to 3b presented before for the amino column. The retention time on Fig. 4a changes from 4.15 to 5.90 min (peak maximum). This leads to V’_p_/V’_a_ varying on average from 0.89 to 1.29 (first moment) and 0.81–1.10 (peak maximum). However, in Fig. 4b, V’_p_/V’_a_ changes between 0.89 and 0.91 (first moment), and 0.81 and 0.83 (peak maximum), which are consistent with uncertainties for this study.

The retention shifts seen in Figs. 3a and 4a are due to the mAbs and lysozyme interactions with AF Formyl particles. For mAb, the major change in retention time/volume was observed first at pH 5.0 and then pH 4.5, which indicated stronger interactions at lower solution pH’s. This highlights the significant risk that protein specific interaction with the column packing can occur in dead columns.

Traditionally for dead volume determinations using a dead column, the NaCl concentration was 0.8 M (23). As it was established that dead volumes could be directly dependent on pH, the effects of NaCl concentration were also evaluated here. Dead volume as a function of NaCl concentration was investigated at a pH 4.5, where mAb and lysozyme indicated the strongest interactions with the column packing.

**Fig. 5:**
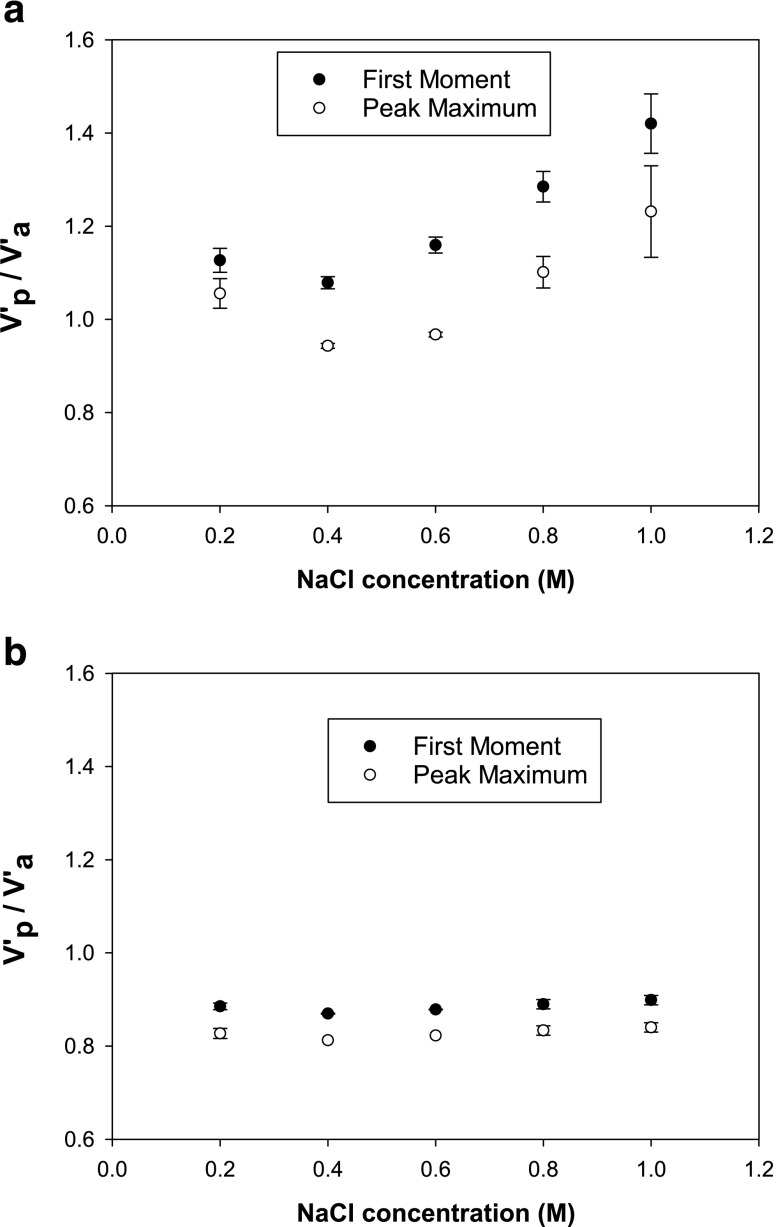
Retention ratios for mAb injections on a Toyopearl AF-Formyl column (**a**) and on a Toyopearl AF-Amino column (**b**) as a function of NaCl concentrations (20 mM sodium acetate pH 4.5).

Figure 5a shows that the NaCl concentration has a significant effect on mAb retention. It has been previously reported that the column matrix (resin particles) could participate in specific interactions with the protein if the salt concentration is too low (31, 41). These interactions can be assumed to occur at a NaCl concentration of 0.2 M or less, where the V’_p_/V’_a_ values are higher. At this concentration there was a large variability in the retention volumes. In Fig. 5a mAb shows that increasing NaCl concentrations results in increasing retention volume variations which has also been reported by other workers (18). In Fig. 5b, the mAb retention does not vary much with NaCl concentration, which also supports the lower level of specific interactions with the Toyopearl AF-Amino columns.

**Fig. 6:**
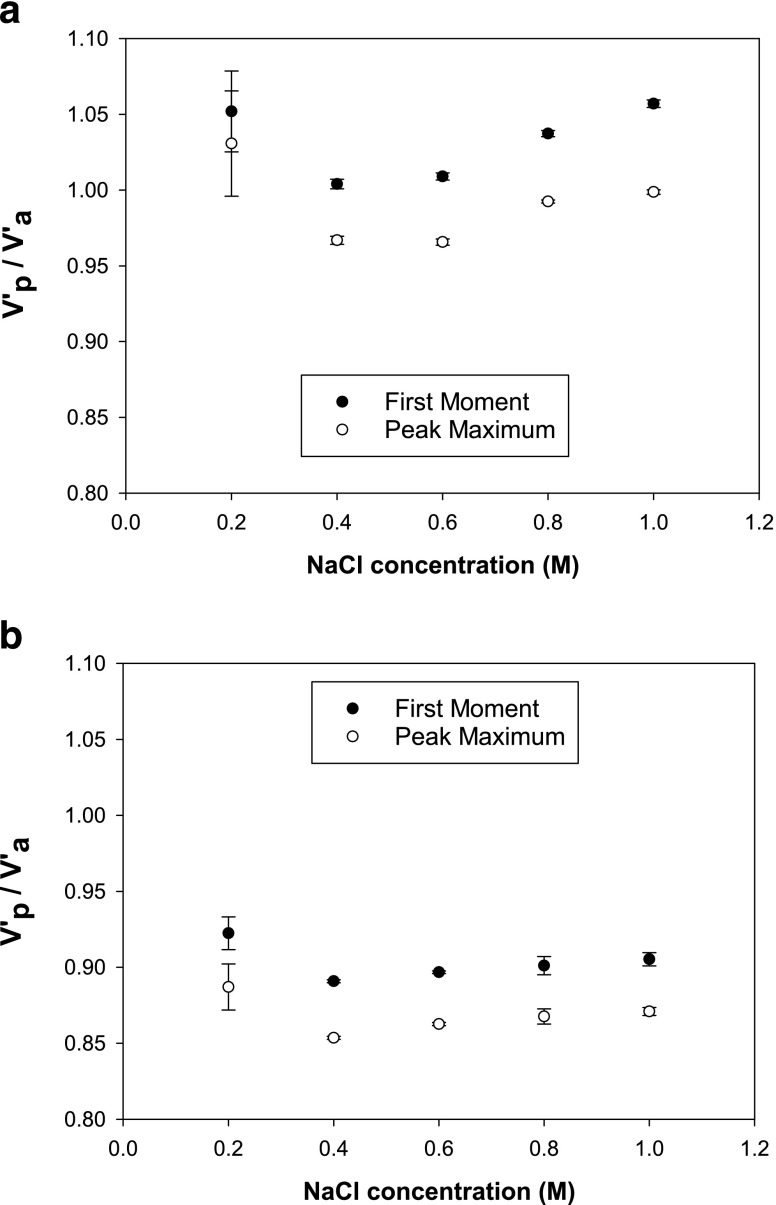
Retention ratios for lysozyme injections on a Toyopearl AF-Formyl column (**a**) and on a Toyopearl AF-Amino column (**b**) as a function of NaCl concentrations (20 mM sodium acetate pH 4.5).

Figure 6a displays the effects of NaCl concentration for lysozyme on a Toyopearl AF-Formyl column. Similarly to Fig. 5a, at a NaCl concentration of 0.2 M or less the interactions are increasing again, possibly due to interactions with the matrix.

The least specific protein-column interactions may be assumed to occur where V’_p_/V’_a_ is the smallest. This occurs at approximately 0.4 M NaCl for both columns with mAbs, and for lysozyme at 0.4–0.6 M. Peak maximum for lysozyme has its minimum at 0.6 M, whereas the first moment analysis give a minimum at 0.4 M.

Usually, the dead volume is measured on a dead column under the same solution conditions as the SIC experiments. However, as revealed in this study, not all dead columns behave in an inert fashion as has been previously presumed.

Therefore, the purpose and effectiveness of using dead columns needs to be carefully considered. If the purpose of the dead column is to allow an accurate dead volume measurement by mimicking a non-interacting molecule of the same hydrodynamic volume of the protein, then many factors such as the column packing, salt concentration and pH need to be taken into account in order to achieve a true non-interacting combination. Here, it is shown that attractive interactions can be prevalent between the protein and the matrix at lower pH’s within certain dead columns. Whether there is also scope for repulsive interactions between the protein and the dead column matrix is harder to determine. If the purpose of the dead volume instead is allow accurate SIC retention data by allowing all interactions with just the matrix to be determined, e.g., if the column is not fully immobilised, then the same column and conditions can be used without taking into account of possible interactions. However, this will not entail an absolute dead volume and for best results many blank or reference experiments will need to be run, one for each condition of the SIC experiment.

### Dead Volume Measurement using a Non-Interacting Probe Molecule

Another established way of estimating the dead volume is to employ a dextran MW standard which is same size as the protein molecule of interest, as the non-interacting molecule. This species is then used with the actual protein immobilised SIC column. For example, a dextran standard of 50 kDa has been established as the same effective size of the mAb allowing evaluation of the dead volume (41). Previously reported dead volumes for mAb are compared with the 50 kDa dextran standard as shown in Table I below. The first column shows the originally measured dead volume for mAb using the Toyopearl AF-Formyl column, sodium acetate buffer pH 4.5, 0.8 M NaCl. The second column includes an optimised dead volume for the same pH sodium acetate buffer as before, but using the Toyopearl AF-Amino column and the NaCl concentration of 0.4 M, with the lowest retention. The third column contains the average dextran/acetone retention of a number of injections of the 50 kDa dextran standard averaged for two mAb immobilised SIC columns under various pH and salt conditions.

**Table I:** Summary for Different Methods to Calculate the Dead Volume

	Dead column method	Dead column method	Dextran method
	AF-Formyl column	AF-Amino column	Dextran 50 kDa
V’_p_/V’_a_ or V_Dextran_/ V_a_	1.285 (±0.033)	0.87 (±0.00)	0.86 (±0.01)
Dead Volume (ml)	2.844	1.918	1.919
k’ (*V*_*p*_ = 2.35 ml)	−0.174 (±0.022)	0.225 (±0.002)	0.225 (±0.009)

Different dextran molecular weight standards were injected into several dead columns such as the AF-Formyl column and the AF-Amino column, as well as a mAb immobilised SIC columns to investigate possible interactions with the matrix. The dextran retention volumes were compared to acetone injections for the different columns to account for any difference in packing characteristics or bed height between the columns. The retention behaviour of the dextran standards were shown to be independent of buffer conditions used and gave consistent results between the two dead columns and the mAb column. This data indicated minimal interactions with the matrix and therefore offers a good alternative method for estimating the dead volume.

The only drawback with using dextran standards is the uncertainty of knowing whether the dextran standard chosen has of the same hydrodynamic volume as the protein of interest. This size was previously estimated using a Superdex 200 GL column by comparing the retention volume of the dextran standards with the retention volume of the mAb, concluding that the 50 kDa dextran standards were the closest size equivalent to a 142 kDa mAb based on the similarity in retention volumes (41). However, the need to estimate the size of the dextran standards and comparing them to the SIC proteins by estimating the retention volume in a size exclusion column means that the use of dextran standards is not necessarily economically advantageous compared to the use of dead columns.

The k’ is calculated using Eq. 3 with V_p_ defined as 2.35 ml.

In Table I the comparisons between the different calculations of the dead volumes demonstrates that a dead volume estimate where specific protein-support interactions are negligible are the same, within experimental error, to the dead volume obtained by the dextran standard method, which leads to approximately the same values for k’. These dead volume results have also been used for calculating the variations in B_2_ as a function of the dead volume estimate used.

**Fig. 7:**
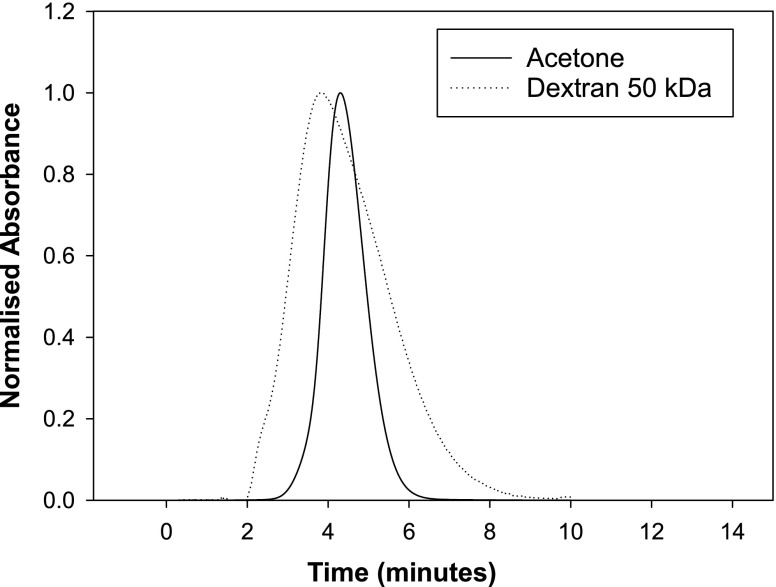
Retention of acetone and dextran 50 kDa on a mAb SIC column.

In Fig. 7 by comparing dextran and acetone retention on a mAb immobilised column it is seen that the dextran standard has a peak maximum and a first moment retention time less than acetone. Protein and polymer peaks are wider than acetone peaks, based on peak width at ½ height basis as noted previously (44).

In addition a simple partitioning model was published by Binabaji *et al*. (41) to determine the protein retention volume for a resin with uniform cylindrical pores:

$$ {V}_{Protein}^{\prime }={V}_{Pore}^{\prime }{\left(1-\frac{r_s}{R}\ \right)}^2+{V}_{Void}^{\prime } $$

Where *r*_*s*_ is the protein radius, *R* is the pore radius, *V*_*Pore*_^′^ is the pore space and *V*_*Void*_^′^ is the interparticle void volume. Binabaji *et al*. (41) defined *r*_*s*_ to be 5.43 nm for the mAb modelled by the dextran standard and R to be 73.9 nm for the resin used in this study. The acetone retention volume per ml of packed resin was determined to be 1.4 ml and is assumed to be equally distributed between the pore space and void volume. For an immobilised protein column of the same size with an average of 32% surface coverage the pore volume accessible is reduced with 0.075 ml and R reduced to 68.47 nm according to the model. This gives a protein retention volume of 1.23 ml and a V_P_/V_a_ of 0.878, confirming the result obtained by the AF-Amino column.

**Fig. 8:**
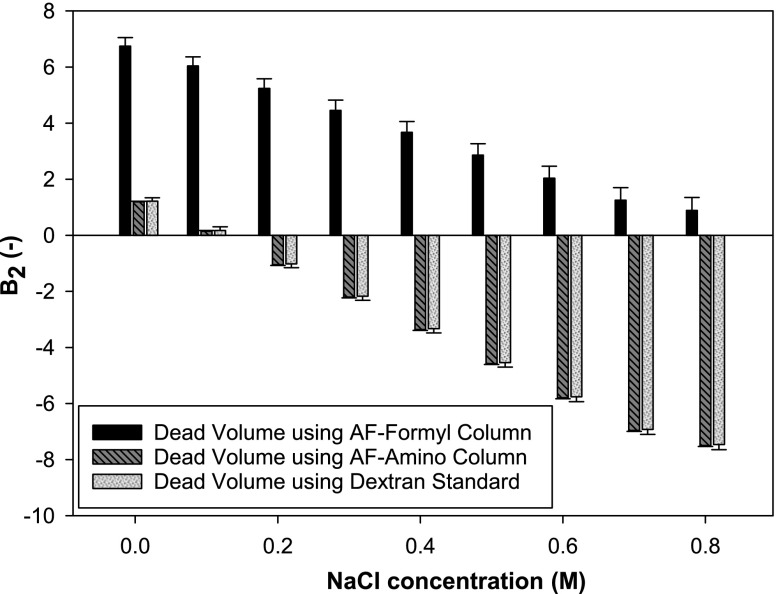
B_2_ values for different NaCl concentrations depending on the dead volume estimates used (20 mM sodium acetate pH 4.5).

The importance of using accurate and robust estimates for the dead volume in determining B_2_ values cannot be over emphasised. Erroneous dead volumes lead directly to inaccurate estimates of B_2_ values. The dead volume estimates from Table I are shown in Fig. 8 where B_2_ of the mAb is determined as a function of increasing NaCl concentrations. The B_2_ estimates in this experiment are decreasing for increasing NaCl concentrations because of increasing charge shielding and therefore subsequent loss of electrostatic stability. However, the actual B_2_ values estimated vary substantially depending upon the dead volume used. Figure 8 provides three different estimated B_2_ values based on the three dead volumes determined in this study, where the second and third dead volumes are very similar (Table I).

As seen in Fig. 8, the increased dead volume estimates using the original AF-Formyl column will lead to only positive B_2_ values being estimated since the protein retention volume is in all the cases less than the dead volume. This result will indicate that only repulsive interactions are present between the mAbs at all NaCl concentrations. The B_2_ values estimated using the optimised dead volume from the AF-Amino column (and 0.4 M NaCl) and from the dextran standard (50 kDa) are the same within experimental error. The error bars displayed are calculated from the standard deviation of dead volumes observed. These B_2_ values indicate, on the contrary, that predominantly attractive interactions between the mAbs occur, apart from at low NaCl concentrations. The difference in B_2_ based on the dead volume ranges from 6 to 9 units depending on the salt concentration, which can lead to a significant error in estimating the B_2_. The use of the dextran standard confirms the result of the dead volume from the AF-Amino dead column as the non-interacting molecule previously seen as a good alternative to dead columns (41).

## Conclusions

The use of a dead column is currently the most common method used to estimate dead volume for SIC. It has been previously assumed that if salt concentrations were ≥0.8 M, any protein-particle interactions present would be negligible for column dead volume measurements. However, in this investigation across a wide range of pH and salt conditions for a number of proteins it has been shown that certain proteins, in this case a mAb, specific interactions with the particles at lower pH values can be significant. The dead volume for a various mAb SIC columns was investigated across a range of different conditions including two different types of dead columns. An optimal dead volume was chosen where the mAb showed the smallest retention with the column packing. This column dead volume was then compared to the dead volume estimated using a non-interacting dextran standard with the same estimated hydrodynamic volume as the mAb. This optimised column dead volume and the dead volume derived from the dextran standard retention proved to be the same within experimental error. This result indicates that using the same type of particles that is generally used as a SIC chromatographic support for a certain protein when immobilised can be an unsuitable choice as a SIC dead column under certain conditions due to specific interaction. Finally, the B_2_ values determined from SIC were compared for the different dead volumes estimates, which highlights the importance of using accurate dead volume estimates in SIC. In general, greater care must be exercised when estimating dead volumes for SIC studies, otherwise substantial errors in virial coefficients may result.

## ABBREVIATIONS

B_2_: Dimensionless Second Virial Coefficient
B_22_: Osmotic Second Virial Coefficient
BSA: Bovine Serum Albumin
LC: Liquid Chromatography
mAb: Monoclonal Antibody
SEC: Size-Exclusion Chromatography
SIC: Self-Interaction Chromatography
